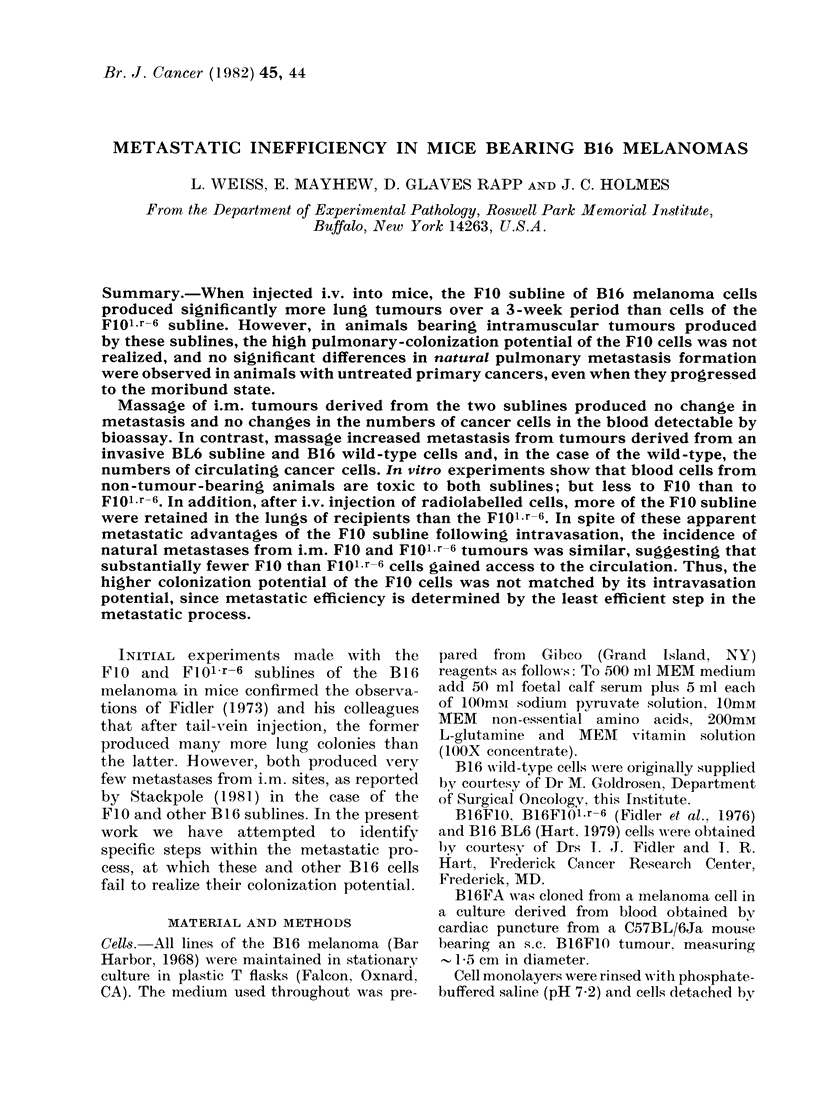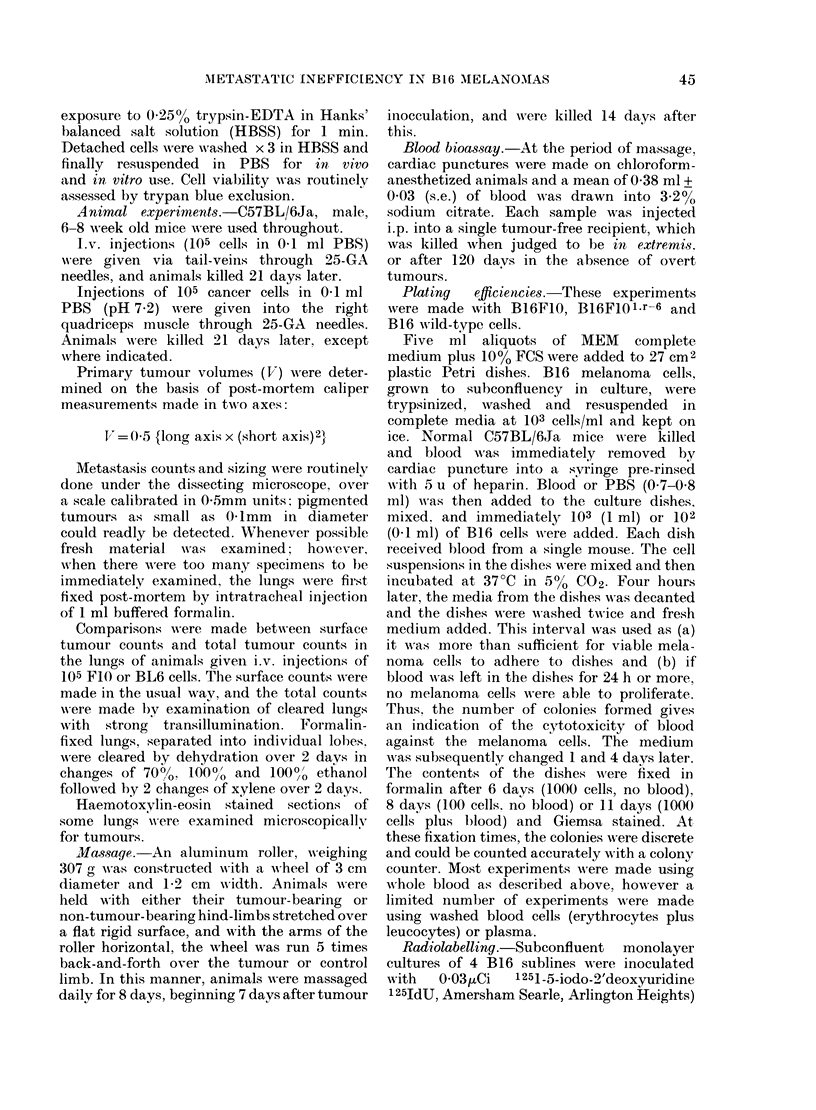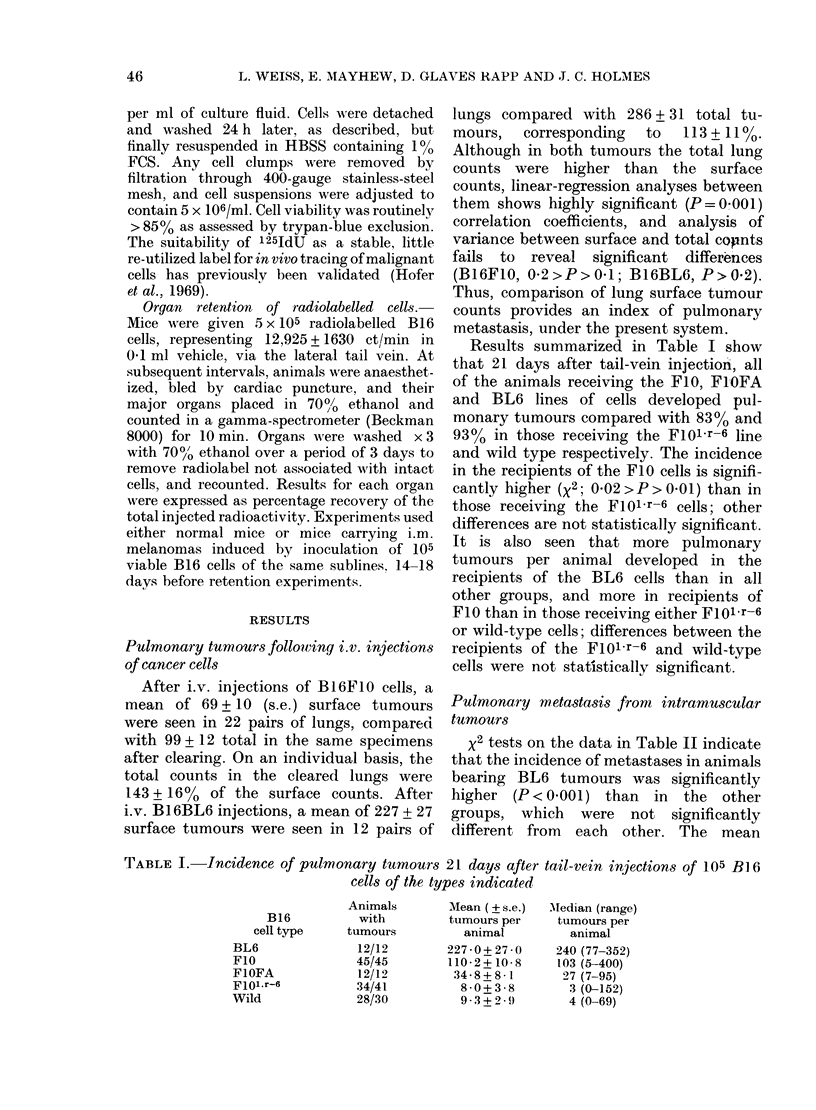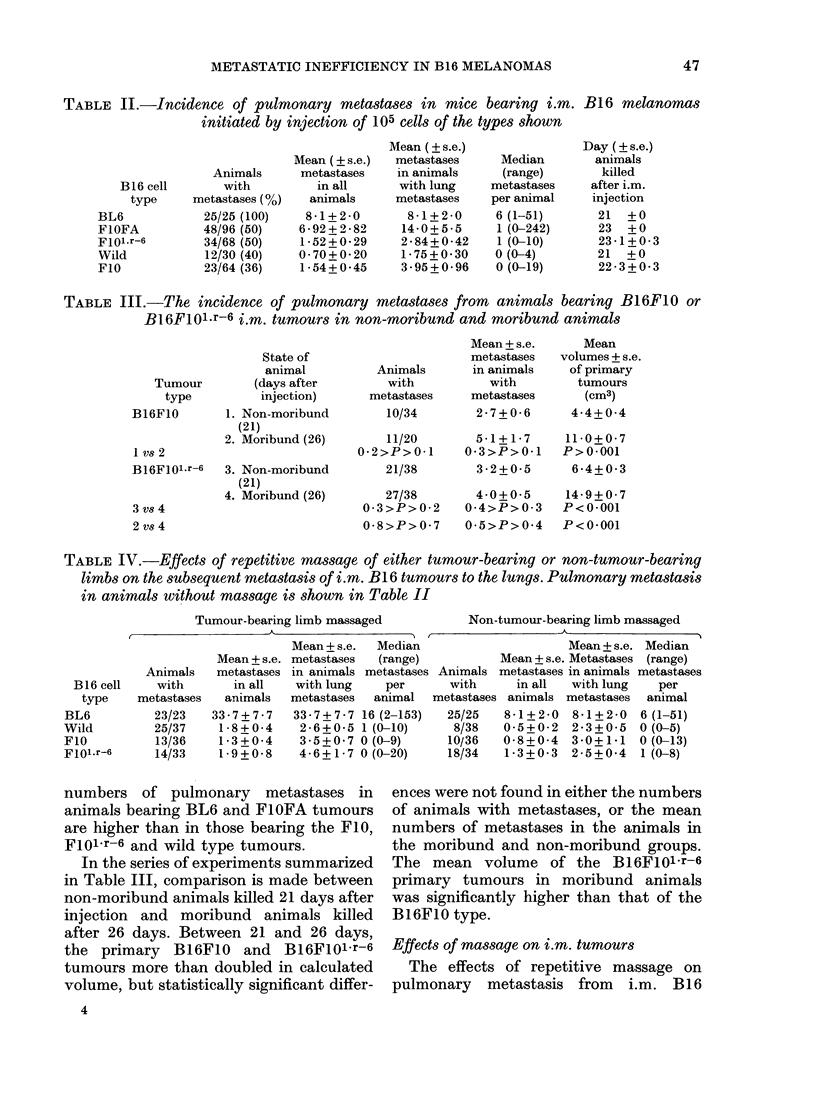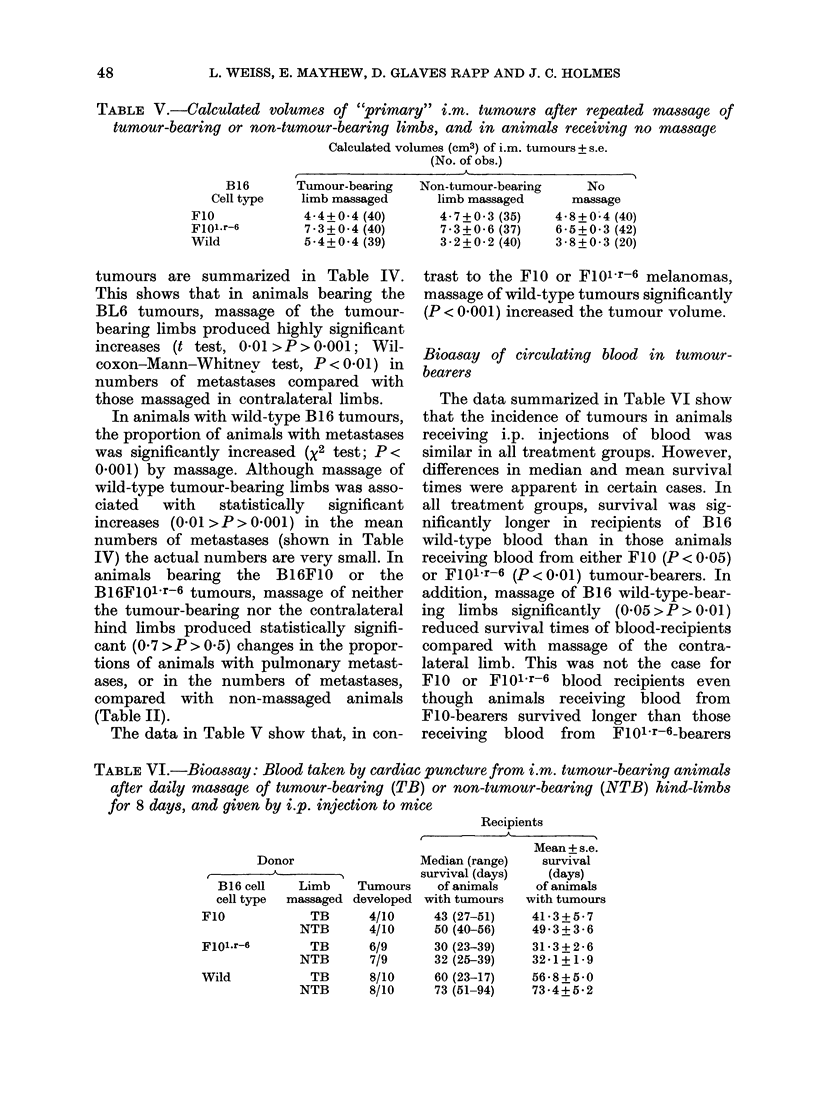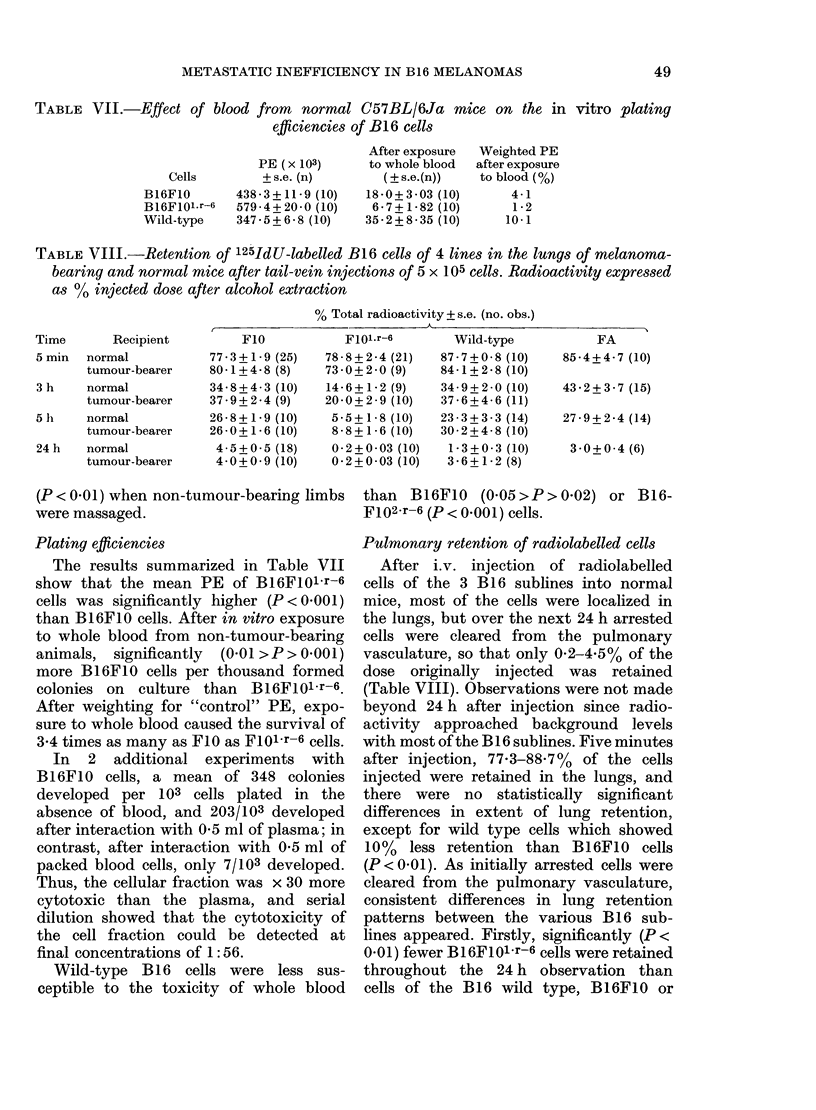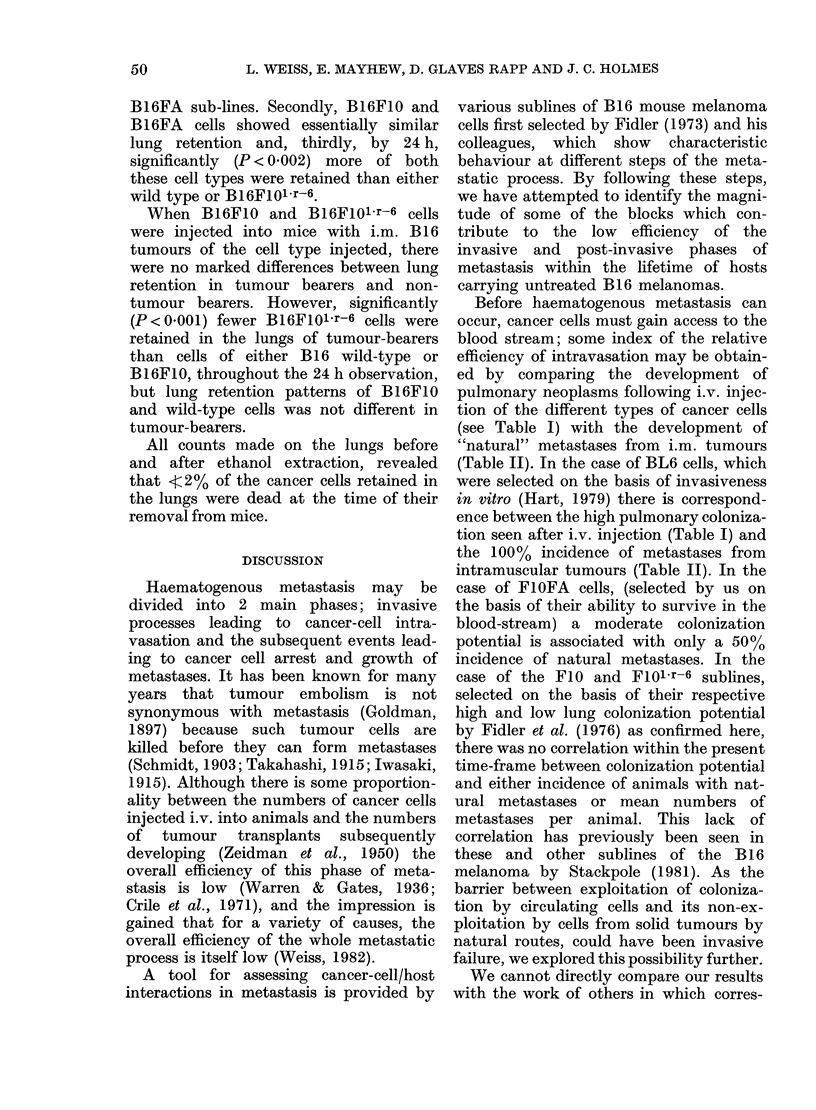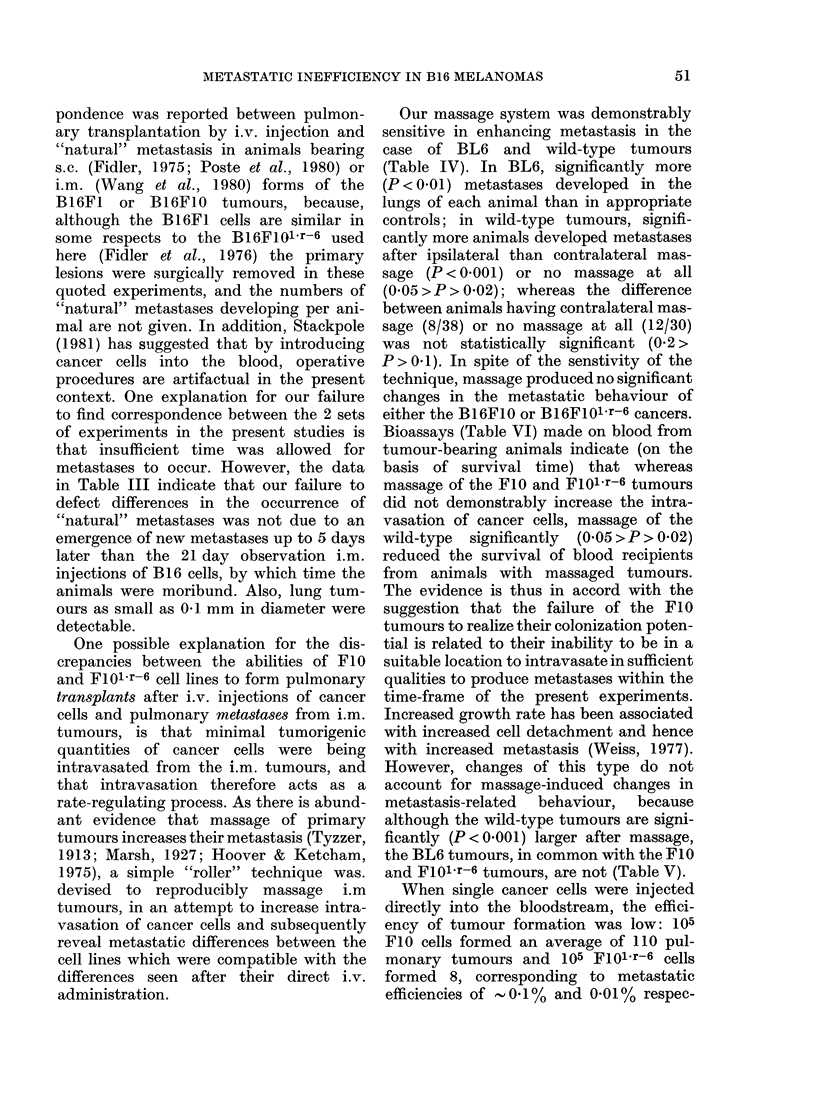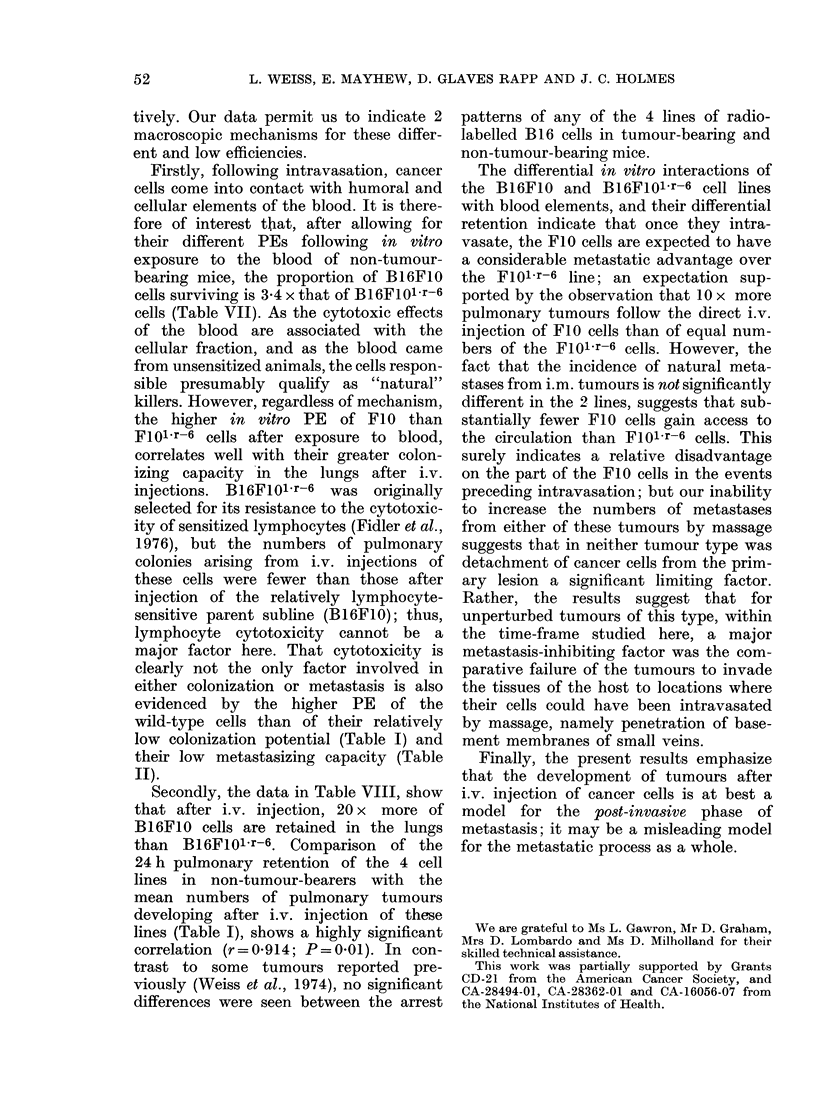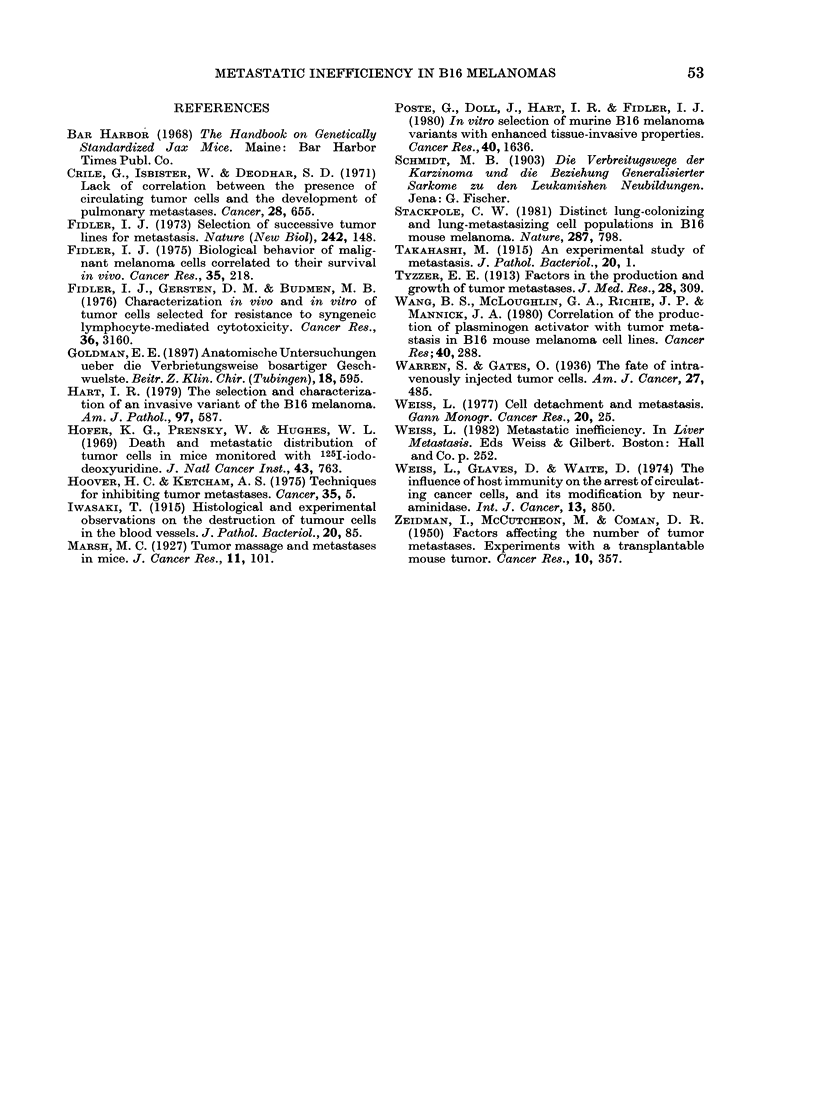# Metastatic inefficiency in mice bearing B16 melanomas.

**DOI:** 10.1038/bjc.1982.6

**Published:** 1982-01

**Authors:** L. Weiss, E. Mayhew, D. G. Rapp, J. C. Holmes

## Abstract

When injected i.v. into mice, the F10 subline of B16 melanoma cells produced significantly more lung tumours over a 3-week period than cells of the F101.r-6 subline. However, in animals bearing intramuscular tumours produced by these sublines, the high pulmonary-colonization potential of the F10 cells was not realized, and no significant differences in natural pulmonary metastasis formation were observed in animals with untreated primary cancers, even when they progressed to the moribund state. Massage of i.m. tumours derived from the two sublines produced no change in metastasis and no changes in the numbers of cancer cells in the blood detectable by bioassay. In contrast, massage increased metastasis from tumours derived from an invasive BL6 subline and B16 wild-type cells and, in the case of the wild-type, the numbers of circulating cancer cells. In vitro experiments show that blood cells from non-tumour-bearing animals are toxic to both sublines; but less to F10 than to F101.r-6. In addition, after i.v. injection of radiolabelled cells, more of the F10 subline were retained in the lungs of recipients than the F101.r-6. In spite of these apparent metastatic advantages of the F10 subline following intravasation, the incidence of natural metastases from i.m. F10 and F101.r-6 tumours was similar, suggesting that substantially fewer F10 than F101.r-6 cells gained access to the circulation. Thus, the higher colonization potential of the F10 cells was not matched by its intravasation potential, since metastatic efficiency is determined by the least efficient step in the metastatic process.


*Br. J. Cancer* (1982) **45**, 44

### METASTATIC INEFFICIENCY IN MICE BEARING B16 MELANOMAS

L. WEISS, E. MAYHEW, D. GLAVES RAPP AND J. C. HOLMES

*From the Department of Experimental Pathology, Roswell Park Memorial Institute, Buffalo, New York* 14263, *U.S.A.*

**Summary.—When injected i.v. into mice, the F10 subline of B16 melanoma cells produced significantly more lung tumours over a 3-week period than cells of the F10$^{1.r-6}$ subline. However, in animals bearing intramuscular tumours produced by these sublines, the high pulmonary-colonization potential of the F10 cells was not realized, and no significant differences in *natural* pulmonary metastasis formation were observed in animals with untreated primary cancers, even when they progressed to the moribund state.**

**Massage of i.m. tumours derived from the two sublines produced no change in metastasis and no changes in the numbers of cancer cells in the blood detectable by bioassay. In contrast, massage increased metastasis from tumours derived from an invasive BL6 subline and B16 wild-type cells and, in the case of the wild-type, the numbers of circulating cancer cells. *In vitro* experiments show that blood cells from non-tumour-bearing animals are toxic to both sublines; but less to F10 than to F10$^{1.r-6}$. In addition, after i.v. injection of radiolabelled cells, more of the F10 subline were retained in the lungs of recipients than the F10$^{1.r-6}$. In spite of these apparent metastatic advantages of the F10 subline following intravasation, the incidence of natural metastases from i.m. F10 and F10$^{1.r-6}$ tumours was similar, suggesting that substantially fewer F10 than F10$^{1.r-6}$ cells gained access to the circulation. Thus, the higher colonization potential of the F10 cells was not matched by its intravasation potential, since metastatic efficiency is determined by the least efficient step in the metastatic process.**

INITIAL experiments made with the F10 and F10$^{1.r-6}$ sublines of the B16 melanoma in mice confirmed the observations of Fidler (1973) and his colleagues that after tail-vein injection, the former produced many more lung colonies than the latter. However, both produced very few metastases from i.m. sites, as reported by Stackpole (1981) in the case of the F10 and other B16 sublines. In the present work we have attempted to identify specific steps within the metastatic process, at which these and other B16 cells fail to realize their colonization potential.

### MATERIAL AND METHODS

*Cells.*—All lines of the B16 melanoma (Bar Harbor, 1968) were maintained in stationary culture in plastic T flasks (Falcon, Oxnard, CA). The medium used throughout was prepared from Gibco (Grand Island, NY) reagents as follows: To 500 ml MEM medium add 50 ml foetal calf serum plus 5 ml each of 100mM sodium pyruvate solution, 10mM MEM non-essential amino acids, 200mM L-glutamine and MEM vitamin solution (100X concentrate).

B16 wild-type cells were originally supplied by courtesy of Dr M. Goldrosen, Department of Surgical Oncology, this Institute.

B16F10, B16F10$^{1.r-6}$ (Fidler *et al.*, 1976) and B16 BL6 (Hart, 1979) cells were obtained by courtesy of Drs I. J. Fidler and I. R. Hart, Frederick Cancer Research Center, Frederick, MD.

B16FA was cloned from a melanoma cell in a culture derived from blood obtained by cardiac puncture from a C57BL/6Ja mouse bearing an s.c. B16F10 tumour, measuring ~1·5 cm in diameter.

Cell monolayers were rinsed with phosphate-buffered saline (pH 7·2) and cells detached by exposure to 0·25% trypsin-EDTA in Hanks' balanced salt solution (HBSS) for 1 min. Detached cells were washed ×3 in HBSS and finally resuspended in PBS for *in vivo* and *in vitro* use. Cell viability was routinely assessed by trypan blue exclusion.

*Animal experiments.*—C57BL/6Ja, male, 6–8 week old mice were used throughout.

I.v. injections ($10^5$ cells in 0·1 ml PBS) were given via tail-veins through 25-GA needles, and animals killed 21 days later.

Injections of $10^5$ cancer cells in 0·1 ml PBS (pH 7·2) were given into the right quadriceps muscle through 25-GA needles. Animals were killed 21 days later, except where indicated.

Primary tumour volumes ($V$) were determined on the basis of post-mortem caliper measurements made in two axes:

$$V = 0{\cdot}5\ \{\text{long axis} \times (\text{short axis})^2\}$$

Metastasis counts and sizing were routinely done under the dissecting microscope, over a scale calibrated in 0·5mm units; pigmented tumours as small as 0·1mm in diameter could readly be detected. Whenever possible fresh material was examined; however, when there were too many specimens to be immediately examined, the lungs were first fixed post-mortem by intratracheal injection of 1 ml buffered formalin.

Comparisons were made between surface tumour counts and total tumour counts in the lungs of animals given i.v. injections of $10^5$ F10 or BL6 cells. The surface counts were made in the usual way, and the total counts were made by examination of cleared lungs with strong transillumination. Formalin-fixed lungs, separated into individual lobes, were cleared by dehydration over 2 days in changes of 70%, 100% and 100% ethanol followed by 2 changes of xylene over 2 days.

Haemotoxylin-eosin stained sections of some lungs were examined microscopically for tumours.

*Massage.*—An aluminum roller, weighing 307 g was constructed with a wheel of 3 cm diameter and 1·2 cm width. Animals were held with either their tumour-bearing or non-tumour-bearing hind-limbs stretched over a flat rigid surface, and with the arms of the roller horizontal, the wheel was run 5 times back-and-forth over the tumour or control limb. In this manner, animals were massaged daily for 8 days, beginning 7 days after tumour inocculation, and were killed 14 days after this.

*Blood bioassay.*—At the period of massage, cardiac punctures were made on chloroform-anesthetized animals and a mean of 0·38 ml ± 0·03 (s.e.) of blood was drawn into 3·2% sodium citrate. Each sample was injected i.p. into a single tumour-free recipient, which was killed when judged to be *in extremis*, or after 120 days in the absence of overt tumours.

*Plating efficiencies.*—These experiments were made with B16F10, $B16F10^{1.r-6}$ and B16 wild-type cells.

Five ml aliquots of MEM complete medium plus 10% FCS were added to 27 $cm^2$ plastic Petri dishes. B16 melanoma cells, grown to subconfluency in culture, were trypsinized, washed and resuspended in complete media at $10^3$ cells/ml and kept on ice. Normal C57BL/6Ja mice were killed and blood was immediately removed by cardiac puncture into a syringe pre-rinsed with 5 u of heparin. Blood or PBS (0·7–0·8 ml) was then added to the culture dishes, mixed, and immediately $10^3$ (1 ml) or $10^2$ (0·1 ml) of B16 cells were added. Each dish received blood from a single mouse. The cell suspensions in the dishes were mixed and then incubated at 37°C in 5% $CO_2$. Four hours later, the media from the dishes was decanted and the dishes were washed twice and fresh medium added. This interval was used as (a) it was more than sufficient for viable melanoma cells to adhere to dishes and (b) if blood was left in the dishes for 24 h or more, no melanoma cells were able to proliferate. Thus, the number of colonies formed gives an indication of the cytotoxicity of blood against the melanoma cells. The medium was subsequently changed 1 and 4 days later. The contents of the dishes were fixed in formalin after 6 days (1000 cells, no blood), 8 days (100 cells, no blood) or 11 days (1000 cells plus blood) and Giemsa stained. At these fixation times, the colonies were discrete and could be counted accurately with a colony counter. Most experiments were made using whole blood as described above, however a limited number of experiments were made using washed blood cells (erythrocytes plus leucocytes) or plasma.

*Radiolabelling.*—Subconfluent monolayer cultures of 4 B16 sublines were inoculated with 0·03$\mu$Ci $^{125}$I-5-iodo-2'deoxyuridine $^{125}$IdU, Amersham Searle, Arlington Heights)

per ml of culture fluid. Cells were detached and washed 24 h later, as described, but finally resuspended in HBSS containing 1% FCS. Any cell clumps were removed by filtration through 400-gauge stainless-steel mesh, and cell suspensions were adjusted to contain $5 \times 10^6$/ml. Cell viability was routinely >85% as assessed by trypan-blue exclusion. The suitability of $^{125}$IdU as a stable, little re-utilized label for *in vivo* tracing of malignant cells has previously been validated (Hofer *et al.*, 1969).

*Organ retention of radiolabelled cells.*— Mice were given $5 \times 10^5$ radiolabelled B16 cells, representing 12,925 ± 1630 ct/min in 0·1 ml vehicle, via the lateral tail vein. At subsequent intervals, animals were anaesthetized, bled by cardiac puncture, and their major organs placed in 70% ethanol and counted in a gamma-spectrometer (Beckman 8000) for 10 min. Organs were washed ×3 with 70% ethanol over a period of 3 days to remove radiolabel not associated with intact cells, and recounted. Results for each organ were expressed as percentage recovery of the total injected radioactivity. Experiments used either normal mice or mice carrying i.m. melanomas induced by inoculation of $10^5$ viable B16 cells of the same sublines, 14–18 days before retention experiments.

### RESULTS

*Pulmonary tumours following i.v. injections of cancer cells*

After i.v. injections of B16F10 cells, a mean of 69 ± 10 (s.e.) surface tumours were seen in 22 pairs of lungs, compared with 99 ± 12 total in the same specimens after clearing. On an individual basis, the total counts in the cleared lungs were 143 ± 16% of the surface counts. After i.v. B16BL6 injections, a mean of 227 ± 27 surface tumours were seen in 12 pairs of lungs compared with 286 ± 31 total tumours, corresponding to 113 ± 11%. Although in both tumours the total lung counts were higher than the surface counts, linear-regression analyses between them shows highly significant ($P = 0{\cdot}001$) correlation coefficients, and analysis of variance between surface and total counts fails to reveal significant differences (B16F10, $0{\cdot}2 > P > 0{\cdot}1$; B16BL6, $P > 0{\cdot}2$). Thus, comparison of lung surface tumour counts provides an index of pulmonary metastasis, under the present system.

Results summarized in Table I show that 21 days after tail-vein injection, all of the animals receiving the F10, F10FA and BL6 lines of cells developed pulmonary tumours compared with 83% and 93% in those receiving the $F10^{l \cdot r-6}$ line and wild type respectively. The incidence in the recipients of the F10 cells is significantly higher ($\chi^2$; $0{\cdot}02 > P > 0{\cdot}01$) than in those receiving the $F10^{l \cdot r-6}$ cells; other differences are not statistically significant. It is also seen that more pulmonary tumours per animal developed in the recipients of the BL6 cells than in all other groups, and more in recipients of F10 than in those receiving either $F10^{l \cdot r-6}$ or wild-type cells; differences between the recipients of the $F10^{l \cdot r-6}$ and wild-type cells were not statistically significant.

*Pulmonary metastasis from intramuscular tumours*

$\chi^2$ tests on the data in Table II indicate that the incidence of metastases in animals bearing BL6 tumours was significantly higher ($P < 0{\cdot}001$) than in the other groups, which were not significantly different from each other. The mean

TABLE I.—*Incidence of pulmonary tumours* 21 *days after tail-vein injections of* $10^5$ *B*16 *cells of the types indicated*

| B16 cell type | Animals with tumours | Mean (± s.e.) tumours per animal | Median (range) tumours per animal |
|---|---|---|---|
| BL6 | 12/12 | 227·0 ± 27·0 | 240 (77–352) |
| F10 | 45/45 | 110·2 ± 10·8 | 103 (5–400) |
| F10FA | 12/12 | 34·8 ± 8·1 | 27 (7–95) |
| $F10^{l \cdot r-6}$ | 34/41 | 8·0 ± 3·8 | 3 (0–152) |
| Wild | 28/30 | 9·3 ± 2·9 | 4 (0–69) |

TABLE II.—*Incidence of pulmonary metastases in mice bearing i.m. B16 melanomas initiated by injection of* $10^5$ *cells of the types shown*

| B16 cell type | Animals with metastases (%) | Mean (± s.e.) metastases in all animals | Mean (± s.e.) metastases in animals with lung metastases | Median (range) metastases per animal | Day (± s.e.) animals killed after i.m. injection |
|---|---|---|---|---|---|
| BL6 | 25/25 (100) | 8·1±2·0 | 8·1±2·0 | 6 (1–51) | 21 ±0 |
| F10FA | 48/96 (50) | 6·92±2·82 | 14·0±5·5 | 1 (0–242) | 23 ±0 |
| $F10^{1.r-6}$ | 34/68 (50) | 1·52±0·29 | 2·84±0·42 | 1 (0–10) | 23·1±0·3 |
| Wild | 12/30 (40) | 0·70±0·20 | 1·75±0·30 | 0 (0–4) | 21 ±0 |
| F10 | 23/64 (36) | 1·54±0·45 | 3·95±0·96 | 0 (0–19) | 22·3±0·3 |

TABLE III.—*The incidence of pulmonary metastases from animals bearing B16F10 or* $B16F10^{1.r-6}$ *i.m. tumours in non-moribund and moribund animals*

| Tumour type | State of animal (days after injection) | Animals with metastases | Mean ± s.e. metastases in animals with metastases | Mean volumes ± s.e. of primary tumours (cm³) |
|---|---|---|---|---|
| B16F10 | 1. Non-moribund (21) | 10/34 | 2·7±0·6 | 4·4±0·4 |
| | 2. Moribund (26) | 11/20 | 5·1±1·7 | 11·0±0·7 |
| 1 *vs* 2 | | $0·2>P>0·1$ | $0·3>P>0·1$ | $P>0·001$ |
| $B16F10^{1.r-6}$ | 3. Non-moribund (21) | 21/38 | 3·2±0·5 | 6·4±0·3 |
| | 4. Moribund (26) | 27/38 | 4·0±0·5 | 14·9±0·7 |
| 3 *vs* 4 | | $0·3>P>0·2$ | $0·4>P>0·3$ | $P<0·001$ |
| 2 *vs* 4 | | $0·8>P>0·7$ | $0·5>P>0·4$ | $P<0·001$ |

TABLE IV.—*Effects of repetitive massage of either tumour-bearing or non-tumour-bearing limbs on the subsequent metastasis of i.m. B16 tumours to the lungs. Pulmonary metastasis in animals without massage is shown in Table II*

| | Tumour-bearing limb massaged | | | | Non-tumour-bearing limb massaged | | | |
|---|---|---|---|---|---|---|---|---|
| B16 cell type | Animals with metastases | Mean ± s.e. metastases in all animals | Mean ± s.e. metastases in animals with lung metastases | Median (range) metastases per animal | Animals with metastases | Mean ± s.e. metastases in all animals | Mean ± s.e. Metastases in animals with lung metastases | Median (range) metastases per animal |
| BL6 | 23/23 | 33·7±7·7 | 33·7±7·7 | 16 (2–153) | 25/25 | 8·1±2·0 | 8·1±2·0 | 6 (1–51) |
| Wild | 25/37 | 1·8±0·4 | 2·6±0·5 | 1 (0–10) | 8/38 | 0·5±0·2 | 2·3±0·5 | 0 (0–5) |
| F10 | 13/36 | 1·3±0·4 | 3·5±0·7 | 0 (0–9) | 10/36 | 0·8±0·4 | 3·0±1·1 | 0 (0–13) |
| $F10^{1.r-6}$ | 14/33 | 1·9±0·8 | 4·6±1·7 | 0 (0–20) | 18/34 | 1·3±0·3 | 2·5±0·4 | 1 (0–8) |

numbers of pulmonary metastases in animals bearing BL6 and F10FA tumours are higher than in those bearing the F10, $F10^{1.r-6}$ and wild type tumours.

In the series of experiments summarized in Table III, comparison is made between non-moribund animals killed 21 days after injection and moribund animals killed after 26 days. Between 21 and 26 days, the primary B16F10 and $B16F10^{1.r-6}$ tumours more than doubled in calculated volume, but statistically significant differences were not found in either the numbers of animals with metastases, or the mean numbers of metastases in the animals in the moribund and non-moribund groups. The mean volume of the $B16F10^{1.r-6}$ primary tumours in moribund animals was significantly higher than that of the B16F10 type.

### *Effects of massage on i.m. tumours*

The effects of repetitive massage on pulmonary metastasis from i.m. B16

TABLE V.—*Calculated volumes of "primary" i.m. tumours after repeated massage of tumour-bearing or non-tumour-bearing limbs, and in animals receiving no massage*

| B16 Cell type | Calculated volumes ($cm^3$) of i.m. tumours ± s.e. (No. of obs.) Tumour-bearing limb massaged | Non-tumour-bearing limb massaged | No massage |
|---|---|---|---|
| F10 | 4·4±0·4 (40) | 4·7±0·3 (35) | 4·8±0·4 (40) |
| $F10^{1.r-6}$ | 7·3±0·4 (40) | 7·3±0·6 (37) | 6·5±0·3 (42) |
| Wild | 5·4±0·4 (39) | 3·2±0·2 (40) | 3·8±0·3 (20) |

tumours are summarized in Table IV. This shows that in animals bearing the BL6 tumours, massage of the tumour-bearing limbs produced highly significant increases (*t* test, $0{\cdot}01 > P > 0{\cdot}001$; Wilcoxon–Mann–Whitney test, $P < 0{\cdot}01$) in numbers of metastases compared with those massaged in contralateral limbs.

In animals with wild-type B16 tumours, the proportion of animals with metastases was significantly increased ($\chi^2$ test; $P < 0{\cdot}001$) by massage. Although massage of wild-type tumour-bearing limbs was associated with statistically significant increases ($0{\cdot}01 > P > 0{\cdot}001$) in the mean numbers of metastases (shown in Table IV) the actual numbers are very small. In animals bearing the B16F10 or the $B16F10^{1\cdot r-6}$ tumours, massage of neither the tumour-bearing nor the contralateral hind limbs produced statistically significant ($0{\cdot}7 > P > 0{\cdot}5$) changes in the proportions of animals with pulmonary metastases, or in the numbers of metastases, compared with non-massaged animals (Table II).

The data in Table V show that, in contrast to the F10 or $F10^{1\cdot r-6}$ melanomas, massage of wild-type tumours significantly ($P < 0{\cdot}001$) increased the tumour volume.

*Bioasay of circulating blood in tumour-bearers*

The data summarized in Table VI show that the incidence of tumours in animals receiving i.p. injections of blood was similar in all treatment groups. However, differences in median and mean survival times were apparent in certain cases. In all treatment groups, survival was significantly longer in recipients of B16 wild-type blood than in those animals receiving blood from either F10 ($P < 0{\cdot}05$) or $F10^{1\cdot r-6}$ ($P < 0{\cdot}01$) tumour-bearers. In addition, massage of B16 wild-type-bearing limbs significantly ($0{\cdot}05 > P > 0{\cdot}01$) reduced survival times of blood-recipients compared with massage of the contralateral limb. This was not the case for F10 or $F10^{1\cdot r-6}$ blood recipients even though animals receiving blood from F10-bearers survived longer than those receiving blood from $F10^{1\cdot r-6}$-bearers

TABLE VI.—*Bioassay: Blood taken by cardiac puncture from i.m. tumour-bearing animals after daily massage of tumour-bearing (TB) or non-tumour-bearing (NTB) hind-limbs for* 8 *days, and given by i.p. injection to mice*

| Donor: B16 cell cell type | Donor: Limb massaged | Tumours developed | Recipients: Median (range) survival (days) of animals with tumours | Recipients: Mean ± s.e. survival (days) of animals with tumours |
|---|---|---|---|---|
| F10 | TB | 4/10 | 43 (27–51) | 41·3±5·7 |
| | NTB | 4/10 | 50 (40–56) | 49·3±3·6 |
| $F10^{1.r-6}$ | TB | 6/9 | 30 (23–39) | 31·3±2·6 |
| | NTB | 7/9 | 32 (25–39) | 32·1±1·9 |
| Wild | TB | 8/10 | 60 (23–17) | 56·8±5·0 |
| | NTB | 8/10 | 73 (51–94) | 73·4±5·2 |

TABLE VII.—*Effect of blood from normal C57BL/6Ja mice on the* in vitro *plating efficiencies of B16 cells*

| Cells | PE (×$10^3$) ±s.e. (n) | After exposure to whole blood (±s.e.(n)) | Weighted PE after exposure to blood (%) |
|---|---|---|---|
| B16F10 | 438·3±11·9 (10) | 18·0±3·03 (10) | 4·1 |
| B16F10$^{1\cdot r-6}$ | 579·4±20·0 (10) | 6·7±1·82 (10) | 1·2 |
| Wild-type | 347·5±6·8 (10) | 35·2±8·35 (10) | 10·1 |

TABLE VIII.—*Retention of* $^{125}$*IdU-labelled B16 cells of 4 lines in the lungs of melanoma-bearing and normal mice after tail-vein injections of* 5 × $10^5$ *cells. Radioactivity expressed as % injected dose after alcohol extraction*

| | | % Total radioactivity ± s.e. (no. obs.) | | | |
|---|---|---|---|---|---|
| Time | Recipient | F10 | F10$^{1\cdot r-6}$ | Wild-type | FA |
| 5 min | normal | 77·3±1·9 (25) | 78·8±2·4 (21) | 87·7±0·8 (10) | 85·4±4·7 (10) |
| | tumour-bearer | 80·1±4·8 (8) | 73·0±2·0 (9) | 84·1±2·8 (10) | |
| 3 h | normal | 34·8±4·3 (10) | 14·6±1·2 (9) | 34·9±2·0 (10) | 43·2±3·7 (15) |
| | tumour-bearer | 37·9±2·4 (9) | 20·0±2·9 (10) | 37·6±4·6 (11) | |
| 5 h | normal | 26·8±1·9 (10) | 5·5±1·8 (10) | 23·3±3·3 (14) | 27·9±2·4 (14) |
| | tumour-bearer | 26·0±1·6 (10) | 8·8±1·6 (10) | 30·2±4·8 (10) | |
| 24 h | normal | 4·5±0·5 (18) | 0·2±0·03 (10) | 1·3±0·3 (10) | 3·0±0·4 (6) |
| | tumour-bearer | 4·0±0·9 (10) | 0·2±0·03 (10) | 3·6±1·2 (8) | |

($P < 0·01$) when non-tumour-bearing limbs were massaged.

*Plating efficiencies*

The results summarized in Table VII show that the mean PE of B16F10$^{1\cdot r-6}$ cells was significantly higher ($P < 0·001$) than B16F10 cells. After *in vitro* exposure to whole blood from non-tumour-bearing animals, significantly ($0·01 > P > 0·001$) more B16F10 cells per thousand formed colonies on culture than B16F10$^{1\cdot r-6}$. After weighting for "control" PE, exposure to whole blood caused the survival of 3·4 times as many as F10 as F10$^{1\cdot r-6}$ cells.

In 2 additional experiments with B16F10 cells, a mean of 348 colonies developed per $10^3$ cells plated in the absence of blood, and 203/$10^3$ developed after interaction with 0·5 ml of plasma; in contrast, after interaction with 0·5 ml of packed blood cells, only 7/$10^3$ developed. Thus, the cellular fraction was ×30 more cytotoxic than the plasma, and serial dilution showed that the cytotoxicity of the cell fraction could be detected at final concentrations of 1:56.

Wild-type B16 cells were less susceptible to the toxicity of whole blood than B16F10 ($0·05 > P > 0·02$) or B16-F10$^{2\cdot r-6}$ ($P < 0·001$) cells.

*Pulmonary retention of radiolabelled cells*

After i.v. injection of radiolabelled cells of the 3 B16 sublines into normal mice, most of the cells were localized in the lungs, but over the next 24 h arrested cells were cleared from the pulmonary vasculature, so that only 0·2–4·5% of the dose originally injected was retained (Table VIII). Observations were not made beyond 24 h after injection since radioactivity approached background levels with most of the B16 sublines. Five minutes after injection, 77·3–88·7% of the cells injected were retained in the lungs, and there were no statistically significant differences in extent of lung retention, except for wild type cells which showed 10% less retention than B16F10 cells ($P < 0·01$). As initially arrested cells were cleared from the pulmonary vasculature, consistent differences in lung retention patterns between the various B16 sublines appeared. Firstly, significantly ($P < 0·01$) fewer B16F10$^{1\cdot r-6}$ cells were retained throughout the 24 h observation than cells of the B16 wild type, B16F10 or

B16FA sub-lines. Secondly, B16F10 and B16FA cells showed essentially similar lung retention and, thirdly, by 24 h, significantly ($P < 0{\cdot}002$) more of both these cell types were retained than either wild type or B16F10$^{l \cdot r - 6}$.

When B16F10 and B16F10$^{l \cdot r - 6}$ cells were injected into mice with i.m. B16 tumours of the cell type injected, there were no marked differences between lung retention in tumour bearers and non-tumour bearers. However, significantly ($P < 0{\cdot}001$) fewer B16F10$^{l \cdot r - 6}$ cells were retained in the lungs of tumour-bearers than cells of either B16 wild-type or B16F10, throughout the 24 h observation, but lung retention patterns of B16F10 and wild-type cells was not different in tumour-bearers.

All counts made on the lungs before and after ethanol extraction, revealed that $\nless 2\%$ of the cancer cells retained in the lungs were dead at the time of their removal from mice.

### DISCUSSION

Haematogenous metastasis may be divided into 2 main phases; invasive processes leading to cancer-cell intravasation and the subsequent events leading to cancer cell arrest and growth of metastases. It has been known for many years that tumour embolism is not synonymous with metastasis (Goldman, 1897) because such tumour cells are killed before they can form metastases (Schmidt, 1903; Takahashi, 1915; Iwasaki, 1915). Although there is some proportionality between the numbers of cancer cells injected i.v. into animals and the numbers of tumour transplants subsequently developing (Zeidman *et al.*, 1950) the overall efficiency of this phase of metastasis is low (Warren & Gates, 1936; Crile *et al.*, 1971), and the impression is gained that for a variety of causes, the overall efficiency of the whole metastatic process is itself low (Weiss, 1982).

A tool for assessing cancer-cell/host interactions in metastasis is provided by various sublines of B16 mouse melanoma cells first selected by Fidler (1973) and his colleagues, which show characteristic behaviour at different steps of the metastatic process. By following these steps, we have attempted to identify the magnitude of some of the blocks which contribute to the low efficiency of the invasive and post-invasive phases of metastasis within the lifetime of hosts carrying untreated B16 melanomas.

Before haematogenous metastasis can occur, cancer cells must gain access to the blood stream; some index of the relative efficiency of intravasation may be obtained by comparing the development of pulmonary neoplasms following i.v. injection of the different types of cancer cells (see Table I) with the development of "natural" metastases from i.m. tumours (Table II). In the case of BL6 cells, which were selected on the basis of invasiveness *in vitro* (Hart, 1979) there is correspondence between the high pulmonary colonization seen after i.v. injection (Table I) and the 100% incidence of metastases from intramuscular tumours (Table II). In the case of F10FA cells, (selected by us on the basis of their ability to survive in the blood-stream) a moderate colonization potential is associated with only a 50% incidence of natural metastases. In the case of the F10 and F10$^{l \cdot r - 6}$ sublines, selected on the basis of their respective high and low lung colonization potential by Fidler *et al.* (1976) as confirmed here, there was no correlation within the present time-frame between colonization potential and either incidence of animals with natural metastases or mean numbers of metastases per animal. This lack of correlation has previously been seen in these and other sublines of the B16 melanoma by Stackpole (1981). As the barrier between exploitation of colonization by circulating cells and its non-exploitation by cells from solid tumours by natural routes, could have been invasive failure, we explored this possibility further.

We cannot directly compare our results with the work of others in which correspondence was reported between pulmonary transplantation by i.v. injection and "natural" metastasis in animals bearing s.c. (Fidler, 1975; Poste *et al.*, 1980) or i.m. (Wang *et al.*, 1980) forms of the B16F1 or B16F10 tumours, because, although the B16F1 cells are similar in some respects to the B16F10$^{l\cdot r-6}$ used here (Fidler *et al.*, 1976) the primary lesions were surgically removed in these quoted experiments, and the numbers of "natural" metastases developing per animal are not given. In addition, Stackpole (1981) has suggested that by introducing cancer cells into the blood, operative procedures are artifactual in the present context. One explanation for our failure to find correspondence between the 2 sets of experiments in the present studies is that insufficient time was allowed for metastases to occur. However, the data in Table III indicate that our failure to defect differences in the occurrence of "natural" metastases was not due to an emergence of new metastases up to 5 days later than the 21 day observation i.m. injections of B16 cells, by which time the animals were moribund. Also, lung tumours as small as 0·1 mm in diameter were detectable.

One possible explanation for the discrepancies between the abilities of F10 and F10$^{l\cdot r-6}$ cell lines to form pulmonary *transplants* after i.v. injections of cancer cells and pulmonary *metastases* from i.m. tumours, is that minimal tumorigenic quantities of cancer cells were being intravasated from the i.m. tumours, and that intravasation therefore acts as a rate-regulating process. As there is abundant evidence that massage of primary tumours increases their metastasis (Tyzzer, 1913; Marsh, 1927; Hoover & Ketcham, 1975), a simple "roller" technique was. devised to reproducibly massage i.m tumours, in an attempt to increase intravasation of cancer cells and subsequently reveal metastatic differences between the cell lines which were compatible with the differences seen after their direct i.v. administration.

Our massage system was demonstrably sensitive in enhancing metastasis in the case of BL6 and wild-type tumours (Table IV). In BL6, significantly more ($P < 0{\cdot}01$) metastases developed in the lungs of each animal than in appropriate controls; in wild-type tumours, significantly more animals developed metastases after ipsilateral than contralateral massage ($P < 0{\cdot}001$) or no massage at all ($0{\cdot}05 > P > 0{\cdot}02$); whereas the difference between animals having contralateral massage (8/38) or no massage at all (12/30) was not statistically significant ($0{\cdot}2 > P > 0{\cdot}1$). In spite of the senstivity of the technique, massage produced no significant changes in the metastatic behaviour of either the B16F10 or B16F10$^{l\cdot r-6}$ cancers. Bioassays (Table VI) made on blood from tumour-bearing animals indicate (on the basis of survival time) that whereas massage of the F10 and F10$^{l\cdot r-6}$ tumours did not demonstrably increase the intravasation of cancer cells, massage of the wild-type significantly ($0{\cdot}05 > P > 0{\cdot}02$) reduced the survival of blood recipients from animals with massaged tumours. The evidence is thus in accord with the suggestion that the failure of the F10 tumours to realize their colonization potential is related to their inability to be in a suitable location to intravasate in sufficient qualities to produce metastases within the time-frame of the present experiments. Increased growth rate has been associated with increased cell detachment and hence with increased metastasis (Weiss, 1977). However, changes of this type do not account for massage-induced changes in metastasis-related behaviour, because although the wild-type tumours are significantly ($P < 0{\cdot}001$) larger after massage, the BL6 tumours, in common with the F10 and F10$^{l\cdot r-6}$ tumours, are not (Table V).

When single cancer cells were injected directly into the bloodstream, the efficiency of tumour formation was low: $10^5$ F10 cells formed an average of 110 pulmonary tumours and $10^5$ F10$^{l\cdot r-6}$ cells formed 8, corresponding to metastatic efficiencies of $\sim 0{\cdot}1\%$ and $0{\cdot}01\%$ respectively. Our data permit us to indicate 2 macroscopic mechanisms for these different and low efficiencies.

Firstly, following intravasation, cancer cells come into contact with humoral and cellular elements of the blood. It is therefore of interest that, after allowing for their different PEs following *in vitro* exposure to the blood of non-tumour-bearing mice, the proportion of B16F10 cells surviving is 3·4 × that of B16F10$^{1 \cdot r-6}$ cells (Table VII). As the cytotoxic effects of the blood are associated with the cellular fraction, and as the blood came from unsensitized animals, the cells responsible presumably qualify as "natural" killers. However, regardless of mechanism, the higher *in vitro* PE of F10 than F10$^{1 \cdot r-6}$ cells after exposure to blood, correlates well with their greater colonizing capacity in the lungs after i.v. injections. B16F10$^{1 \cdot r-6}$ was originally selected for its resistance to the cytotoxicity of sensitized lymphocytes (Fidler *et al.*, 1976), but the numbers of pulmonary colonies arising from i.v. injections of these cells were fewer than those after injection of the relatively lymphocyte-sensitive parent subline (B16F10); thus, lymphocyte cytotoxicity cannot be a major factor here. That cytotoxicity is clearly not the only factor involved in either colonization or metastasis is also evidenced by the higher PE of the wild-type cells than of their relatively low colonization potential (Table I) and their low metastasizing capacity (Table II).

Secondly, the data in Table VIII, show that after i.v. injection, 20 × more of B16F10 cells are retained in the lungs than B16F10$^{1 \cdot r-6}$. Comparison of the 24 h pulmonary retention of the 4 cell lines in non-tumour-bearers with the mean numbers of pulmonary tumours developing after i.v. injection of these lines (Table I), shows a highly significant correlation ($r = 0 \cdot 914$; $P = 0 \cdot 01$). In contrast to some tumours reported previously (Weiss *et al.*, 1974), no significant differences were seen between the arrest patterns of any of the 4 lines of radio-labelled B16 cells in tumour-bearing and non-tumour-bearing mice.

The differential *in vitro* interactions of the B16F10 and B16F10$^{1 \cdot r-6}$ cell lines with blood elements, and their differential retention indicate that once they intravasate, the F10 cells are expected to have a considerable metastatic advantage over the F10$^{1 \cdot r-6}$ line; an expectation supported by the observation that 10 × more pulmonary tumours follow the direct i.v. injection of F10 cells than of equal numbers of the F10$^{1 \cdot r-6}$ cells. However, the fact that the incidence of natural metastases from i.m. tumours is *not* significantly different in the 2 lines, suggests that substantially fewer F10 cells gain access to the circulation than F10$^{1 \cdot r-6}$ cells. This surely indicates a relative disadvantage on the part of the F10 cells in the events preceding intravasation; but our inability to increase the numbers of metastases from either of these tumours by massage suggests that in neither tumour type was detachment of cancer cells from the primary lesion a significant limiting factor. Rather, the results suggest that for unperturbed tumours of this type, within the time-frame studied here, a major metastasis-inhibiting factor was the comparative failure of the tumours to invade the tissues of the host to locations where their cells could have been intravasated by massage, namely penetration of basement membranes of small veins.

Finally, the present results emphasize that the development of tumours after i.v. injection of cancer cells is at best a model for the *post-invasive* phase of metastasis; it may be a misleading model for the metastatic process as a whole.

We are grateful to Ms L. Gawron, Mr D. Graham, Mrs D. Lombardo and Ms D. Milholland for their skilled technical assistance.

This work was partially supported by Grants CD-21 from the American Cancer Society, and CA-28494-01, CA-28362-01 and CA-16056-07 from the National Institutes of Health.

### REFERENCES

BAR HARBOR (1968) *The Handbook on Genetically Standardized Jax Mice*. Maine: Bar Harbor Times Publ. Co.

CRILE, G., ISBISTER, W. & DEODHAR, S. D. (1971) Lack of correlation between the presence of circulating tumor cells and the development of pulmonary metastases. *Cancer*, **28**, 655.

FIDLER, I. J. (1973) Selection of successive tumor lines for metastasis. *Nature* (*New Biol*), **242**, 148.

FIDLER, I. J. (1975) Biological behavior of malignant melanoma cells correlated to their survival *in vivo*. *Cancer Res.*, **35**, 218.

FIDLER, I. J., GERSTEN, D. M. & BUDMEN, M. B. (1976) Characterization *in vivo* and *in vitro* of tumor cells selected for resistance to syngeneic lymphocyte-mediated cytotoxicity. *Cancer Res.*, **36**, 3160.

GOLDMAN, E. E. (1897) Anatomische Untersuchungen ueber die Verbrietungsweise bosartiger Geschwuelste. *Beitr. Z. Klin. Chir.* (*Tubingen*), **18**, 595.

HART, I. R. (1979) The selection and characterization of an invasive variant of the B16 melanoma. *Am. J. Pathol.*, **97**, 587.

HOFER, K. G., PRENSKY, W. & HUGHES, W. L. (1969) Death and metastatic distribution of tumor cells in mice monitored with $^{125}$I-iododeoxyuridine. *J. Natl Cancer Inst.*, **43**, 763.

HOOVER, H. C. & KETCHAM, A. S. (1975) Techniques for inhibiting tumor metastases. *Cancer*, **35**, 5.

IWASAKI, T. (1915) Histological and experimental observations on the destruction of tumour cells in the blood vessels. *J. Pathol. Bacteriol.*, **20**, 85.

MARSH, M. C. (1927) Tumor massage and metastases in mice. *J. Cancer Res.*, **11**, 101.

POSTE, G., DOLL, J., HART, I. R. & FIDLER, I. J. (1980) *In vitro* selection of murine B16 melanoma variants with enhanced tissue-invasive properties. *Cancer Res.*, **40**, 1636.

SCHMIDT, M. B. (1903) *Die Verbreitugswege der Karzinoma und die Beziehung Generalisierter Sarkome zu den Leukamishen Neubildungen.* Jena: G. Fischer.

STACKPOLE, C. W. (1981) Distinct lung-colonizing and lung-metastasizing cell populations in B16 mouse melanoma. *Nature*, **287**, 798.

TAKAHASHI, M. (1915) An experimental study of metastasis. *J. Pathol. Bacteriol.*, **20**, 1.

TYZZER, E. E. (1913) Factors in the production and growth of tumor metastases. *J. Med. Res.*, **28**, 309.

WANG, B. S., MCLOUGHLIN, G. A., RICHIE, J. P. & MANNICK, J. A. (1980) Correlation of the production of plasminogen activator with tumor metastasis in B16 mouse melanoma cell lines. *Cancer Res*; **40**, 288.

WARREN, S. & GATES, O. (1936) The fate of intravenously injected tumor cells. *Am. J. Cancer*, **27**, 485.

WEISS, L. (1977) Cell detachment and metastasis. *Gann Monogr. Cancer Res.*, **20**, 25.

WEISS, L. (1982) Metastatic inefficiency. In *Liver Metastasis*. Eds Weiss & Gilbert. Boston: Hall and Co. p. 252.

WEISS, L., GLAVES, D. & WAITE, D. (1974) The influence of host immunity on the arrest of circulating cancer cells, and its modification by neuraminidase. *Int. J. Cancer*, **13**, 850.

ZEIDMAN, I., MCCUTCHEON, M. & COMAN, D. R. (1950) Factors affecting the number of tumor metastases. Experiments with a transplantable mouse tumor. *Cancer Res.*, **10**, 357.